# Cross-Sectional analysis of demographic and clinical characteristics of patients in the United States using icosapent ethyl

**DOI:** 10.3389/fcvm.2025.1411233

**Published:** 2025-03-14

**Authors:** Peter P. Toth, John R. Nelson, Handrean Soran, Om P. Ganda, Nathan D. Wong, Hakima Hannachi, David Abrahamson, Josh Hartman, Sierra Luciano, Sephy Philip

**Affiliations:** ^1^ CGH Medical Center, Sterling, IL, United States; ^2^ Cicarrone Center for the Prevention of Cardiovascular Disease, Johns Hopkins University School of Medicine, Baltimore, MD, United States; ^3^ California Cardiovascular Institute, Fresno, CA, United States; ^4^ Department of Endocrinology, Diabetes, and Metabolism, Manchester University Hospital NHS Foundation Trust, Manchester, United Kingdom; ^5^ Clinical Research and Adult Diabetes Sections, Department of Medicine, Joslin Diabetes Center and Harvard Medical School, Boston, MA, United States; ^6^ Division of Cardiology, University of California, Irvine, CA, United States; ^7^ Department of Medical Affairs, Amarin Pharma Inc., Zug, Switzerland; ^8^ Department of Medical Affairs, Amarin Pharma Inc., Bridgewater, NJ, United States; ^9^ Trial Design & Optimization, TriNetX, Cambridge, MA, United States

**Keywords:** hypertriglyceridemia, triglyceride, cardiovascular disease, diabetes mellitus, statin, icosapent ethyl

## Abstract

**Introduction:**

Icosapent ethyl (IPE) is indicated for the treatment of severe hypertriglyceridemia (triglycerides ≥500 mg/dl) and for reducing the risk of cardiovascular (CV) events in statin-treated adults with moderately elevated triglycerides (150–499 mg/dl) and established CV disease [secondary prevention (SP)] or diabetes with CV risk factors [primary prevention (PP)]. We describe real-world characteristics of US patients taking IPE.

**Methods:**

Patients with ≥2 IPE prescriptions were identified in the TriNetX database. PP criteria were: ≥50 years with diabetes mellitus, ≥1 additional CV risk factor, and triglycerides 150–499 mg/dl. SP criteria were established CV disease and triglycerides 150–499 mg/dl.

**Results:**

Among patients with ≥2 IPE prescriptions and triglyceride data, 56.2% (18,897/33,645) met PP or SP criteria, 28.0% (9,431/33,645) had severe hypertriglyceridemia. In the PP and SP cohorts, mean (SD) ages were 62.7 (8.0) and 64.0 (10.7) years, respectively. In the SP cohort, coronary artery disease was the most common pre-existing CV disease (85.8%) and many had diabetes (63.1%). In the PP and SP cohorts, 81.7% and 90.4%, respectively, received statin treatment. Before IPE initiation, mean (SD; median) triglyceride levels were 305 (150; 253) and 279 (142; 230) mg/dl in the PP and SP cohorts, respectively, and mean/median LDL-C levels were <100 mg/dl in both.

**Discussion:**

Patients taking IPE had characteristics consistent with its indication, including well-controlled LDL-C levels with statin use. The higher triglyceride levels before IPE initiation suggest that IPE may be underutilized in patients at high risk for CV events; however, future studies are needed.

## 1 Introduction

Icosapent ethyl (IPE) is a purified ethyl ester of eicosapentaenoic acid indicated in the United States for the treatment of severe hypertriglyceridemia [triglyceride (TG) levels ≥500 mg/dl] (1). IPE is also approved in the United States and other countries for reducing the risk of cardiovascular (CV) events in adults taking a statin who have moderately elevated TG levels (150–499 mg/dl) and either established CV disease or diabetes mellitus with additional CV risk factors (1–4).

The pivotal Reduction of Cardiovascular Events With EPA – Intervention Trial (REDUCE-IT) evaluated the efficacy of IPE in reducing CV events in patients ≥45 years of age with established CV disease or ≥50 years of age with diabetes and at least one additional CV risk factor and with moderately elevated TG levels (150–499 mg/dl) at baseline (5). Additional inclusion criteria were baseline low-density lipoprotein cholesterol (LDL-C) of 41–100 mg/dl and stable statin use for ≥4 weeks. Results from this trial showed that IPE-treated patients had a 25% risk reduction in major CV events and a 30% risk reduction in total events compared with those taking placebo (*P* < 0.001 for both) (5, 6).

Real-world data from the National Health and Nutrition Examination Survey indicated that, between 1999 and 2016, approximately 3 million US adults may have had demographic and baseline clinical characteristics that match the inclusion criteria for REDUCE-IT (7). Real-world data on the characteristics of patients who receive IPE are lacking. Therefore, we aimed to describe the real-world demographic and clinical characteristics of patients in the United States who are taking IPE and meeting primary (PP) or secondary prevention (SP) criteria.

## 2 Methods

### 2.1 Study population

This cross-sectional, observational study included patients who were identified in the TriNetX database, which contains electronic medical record (EMR) data from >100 million patients across 61 healthcare organizations in the United States. Included patients had at least two IPE prescriptions on two separate dates; there were no other inclusion or exclusion criteria, including no restrictions on calendar date or sex. Data presented are from patients satisfying the inclusion criteria on January 17, 2024. Included patient data were divided into PP and SP cohorts. The PP cohort included patients with two or more IPE prescriptions, aged ≥50 years with diabetes mellitus, evidence of one or more additional CV risk factor, and TG levels 150–499 mg/dl. CV risk factors included history of cigarette smoking; hypertension or an antihypertensive medication; high-density lipoprotein cholesterol (HDL-C) level ≤40 mg/dl; high-sensitivity C-reactive protein concentration >3.00 mg/L (0.3 mg/dl); renal dysfunction; retinopathy; microalbuminuria or macroalbuminuria; and ankle-brachial index <0.9. Patients with coronary artery disease, cerebrovascular disease, carotid artery disease, or peripheral arterial disease were excluded from this group. The SP cohort included patients with two or more IPE prescriptions with established coronary artery disease, cerebrovascular disease, carotid artery disease, or peripheral arterial disease, and TG levels 150–499 mg/dl.

### 2.2 Data collection

Demographic and clinical characteristics collected from the TriNetX database included age, sex, race, ethnicity, body mass index, and ankle-brachial index. Prior or coexisting conditions and medications were also collected and included diabetes, diabetes-related disease, history of CV disease, other comorbidities, and hypolipidemic, CV-related, and antidiabetic medications; these were captured from any time on or before the first instance of IPE in the EMR. Laboratory values were reported from measurements taken the same day to 3 months before the first instance of IPE in the EMR and included hemoglobin A1c, LDL-C, TG, HDL-C, high-sensitivity C-reactive protein, indices of albuminuria, and creatinine clearance to determine renal function.

## 3 Results

**Figure 1:**
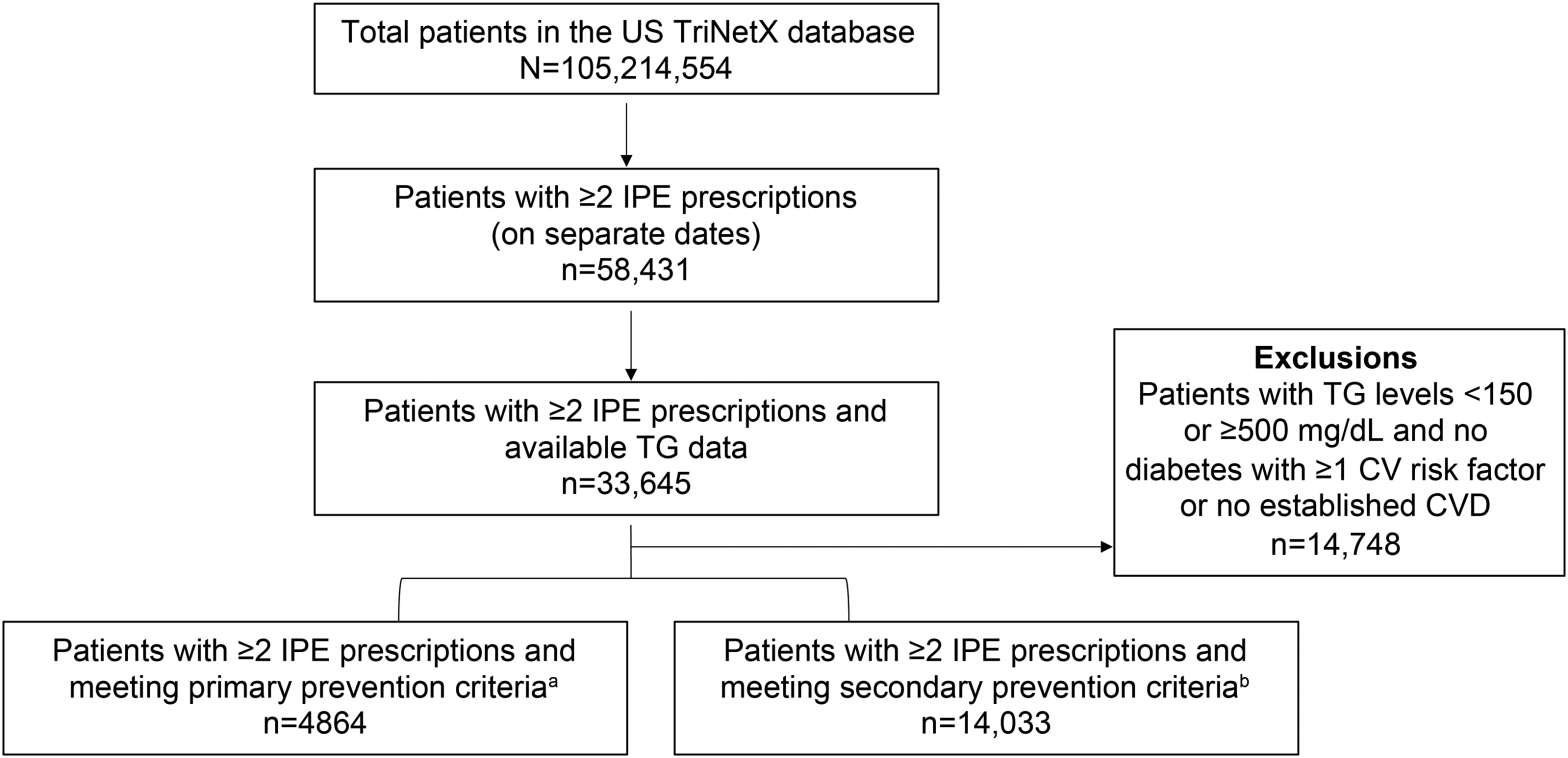
Patient attrition. CV, cardiovascular; CVD, cardiovascular disease; IPE, icosapent ethyl; TG, triglyceride; US, United States. ^a^Patients aged ≥50 years with diabetes mellitus, evidence of ≥1 additional CV risk factor, and TG level 150–499 mg/dl. ^b^Patients with established coronary artery disease, cerebrovascular disease, carotid artery disease, or peripheral arterial disease and TG level 150–499 mg/dl.

**Figure 2:**
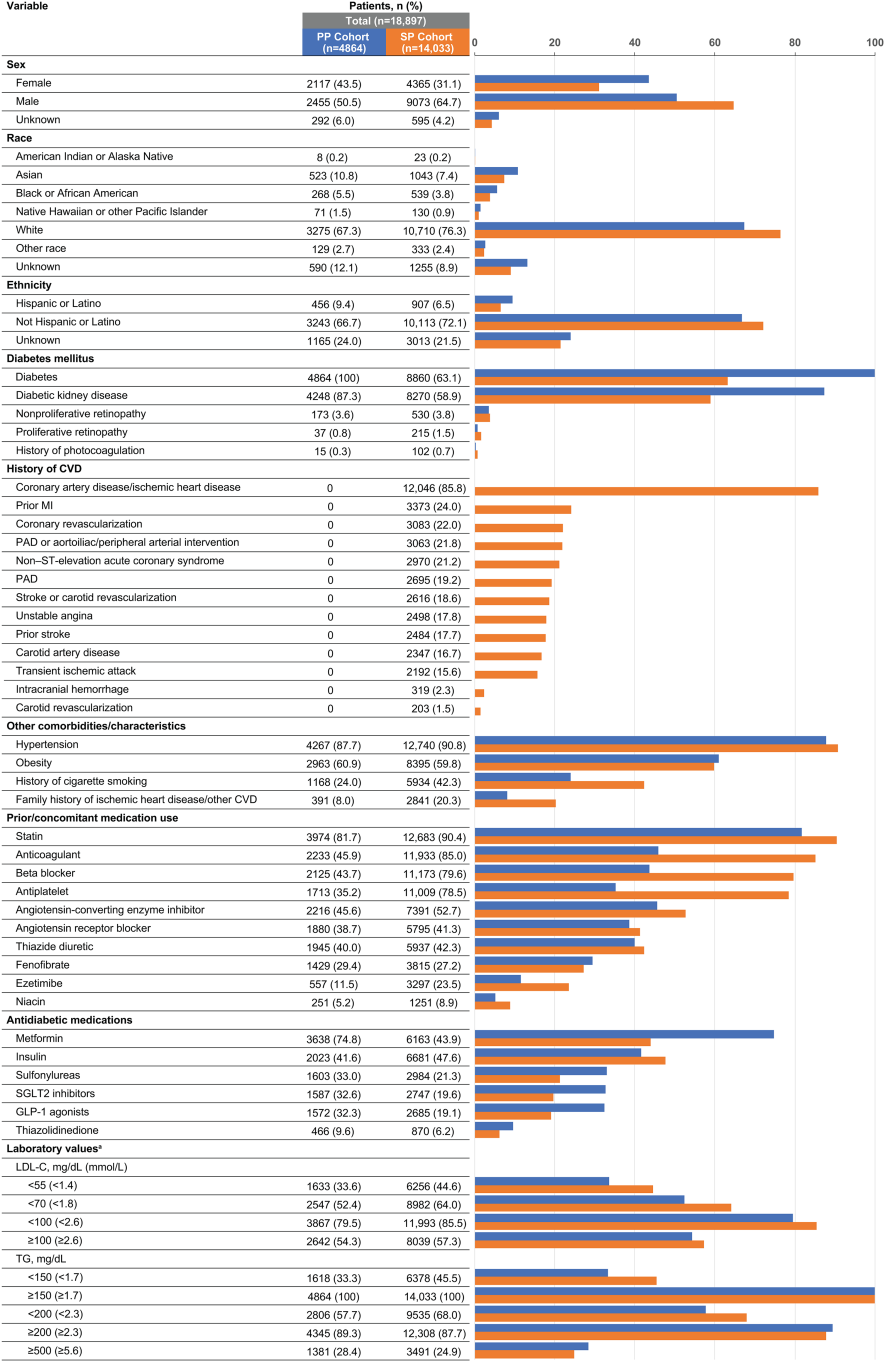
Demographic and clinical characteristics of US patients taking icosapent ethyl and meeting primary or secondary prevention criteria. CVD, cardiovascular disease; GLP-1, glucagon-like peptide; LDL-C, low-density lipoprotein cholesterol; MI, myocardial infarction; PAD, peripheral arterial disease; PP, primary prevention; SGLT2, sodium-glucose cotransporter 2; SP, secondary prevention; TG, triglyceride; US, United States. ^a^Percentages for laboratory value categories add up to more than 100 due to the inclusion of multiple samples per patient.

Of the total number of patients with two or more IPE prescriptions and available TG data, 56.2% (18,897/33,645) met PP or SP criteria (Figure 1). Among the total number of patients with two or more IPE prescriptions and available TG data, 28.0% had a TG level ≥500 mg/dl before initiating IPE. In the PP and SP cohorts, mean (SD) ages were 62.7 (8.0) and 64.0 (10.7) years and mean (SD) body mass indices were 32.9 (5.9) and 32.0 (5.7) kg/m^2^, respectively. The majority of patients in both prevention groups were White (PP cohort: 67.3%; SP cohort: 76.3%) and not Hispanic or Latino (PP cohort: 66.7%; SP cohort: 72.1%; Figure 2).

The prevalences of risk factors that qualified patients for the PP cohort were: hypertension or an antihypertensive medication, 95%; HDL-C < 40 mg/dl, 80.7%; micro- or macroalbuminuria, 47.7%; history of cigarette smoking, 24%; nonproliferative retinopathy, 3.6%; high-sensitivity C-reactive protein ≥3.0 mg/L, 3.5%; proliferative retinopathy, 0.8%; moderate renal dysfunction (creatinine clearance 30–59 ml/min), 0.3%; and ankle-brachial index ≤0.9, 0%. In the SP cohort, coronary artery disease (85.8%) was the most common pre-existing CV disease (Figure 2). Many patients in the SP cohort also had diabetes (63.1%); 43.9% of patients in this cohort were taking metformin (vs. 74.8% in the PP cohort) and 47.6% were taking insulin (vs. 41.6% in the PP cohort; Figure 2). The mean (SD) hemoglobin A1c was similar between cohorts; 7.0% (1.6%) in the SP cohort and 7.4% (1.4%) in the PP cohort.

The prevalence of statin treatment in the PP cohort was 81.7% and in the SP cohort was 90.4% (Figure 2). Additionally, patients had evidence of other cholesterol-lowering medications including ezetimibe (11.5% PP; 23.5% SP), bile acid sequestrants (4.2% PP; 5.0% SP), PCSK9 inhibitors (1.2% PP; 8.4% SP), and bempedoic acid (0.1% PP; 0.3% SP). Similar percentages of patients in the PP and SP cohorts concomitantly used fenofibrate (29.4% and 27.2%, respectively) or niacin (5.2% and 8.9%, respectively; Figure 2). In the 3 months before IPE initiation, mean (SD; median) TG levels were 305 (150; 253) and 279 (142; 230) mg/dl in the PP and SP cohorts, respectively, and mean/median LDL-C levels were <100 mg/dl in both cohorts [mean (SD; median), 89.6 (34.2; 84.5) and 86.9 (34.0; 79.5) mg/dl in the PP and SP cohorts, respectively].

## 4 Discussion

In this large cross-sectional study utilizing one of the largest and most comprehensive EMR databases available in the United States, patients taking IPE had clinical characteristics that were consistent with IPE's indication. Patients in both the PP and SP cohorts had median LDL-C < 100 mg/dl, were taking statins, and had elevated TG levels before initiating IPE. The most common risk factor in the PP cohort was hypertension, and a majority of patients in the SP cohort had coronary heart disease. The results from this study add to the limited real-world data on IPE use and expand on results from a previous cross-sectional study that identified US adults from the National Health and Nutrition Examination Survey from 1999 to 2016 who met REDUCE-IT inclusion criteria (7). The authors estimated that approximately 3 million patients in the US are eligible for IPE therapy based on the inclusion criteria from REDUCE-IT and 4.5 million may be eligible based on the FDA label; approximately 1.1 and 1.9 million eligible patients were estimated to meet PP and SP criteria, respectively.

Consistent with the indication for IPE, the prevalence of statin treatment in both cohorts was high, and similar percentages of patients in both cohorts were using other TG-lowering medications which have not been shown to reduce CV events in high-risk patients (8, 9). In this study, patients in both the PP and SP cohorts had high mean/median TG levels before starting IPE. Results from REDUCE-IT showed reductions in CV events in patients taking IPE regardless of baseline TG levels (5). Taken together with our real-world results, this suggests that IPE may be underutilized for CV risk reduction in patients with more moderately elevated TG levels (e.g., 150–499 mg/dl) and at high risk for CV events. Future studies are needed to determine the real-world rate of IPE use among those who are eligible for treatment.

A strength of this study was the inclusion and analysis of over 100 million electronic medical records across multiple health organizations in the United States from the TriNetX database, among the largest available. However, as all EMR databases include patients who have sought medical care for numerous reasons, the study sample may not be fully representative of the US population. Additionally, there may be incomplete patient records and missing data if a patient seeks care at a health provider outside of organizations included in the database. As the TriNetX database relies on billing codes, it may not accurately capture the exact timing of events. A further limitation involved the TriNetX analysis platform, which did not allow application of sex-specific HDL-C or age cut points as used in REDUCE-IT, which limited the ability to use those cut points to define eligibility in the PP cohort.

## 5 Conclusion

In summary, this study demonstrated that real-world IPE use in patients in the United States is consistent with its label. Patients taking IPE in both the PP and SP cohorts had well-controlled LDL-C levels and were taking statins. The high baseline mean/median TG levels in PP and SP groups may indicate that IPE is underutilized in patients at high risk for CV events; however, future studies are needed to quantify the rate of IPE use among eligible patients.

## Data Availability

The datasets presented in this article are not readily available because they are proprietary. Individual participant data underlying the results reported in this publication, along with a data dictionary, may be made available to approved investigators for secondary analyses for a fee from TriNetX. Requests to access the datasets should be directed to sephy.philip@amarincorp.com.
